# Maternal socialization goals and the spontaneous prosocial behavior of children in rural contexts

**DOI:** 10.1186/s41155-018-0108-x

**Published:** 2018-10-19

**Authors:** Bianca Reis Fonseca, Lília Iêda Chaves Cavalcante, Joscha Kärtner, Moritz Köster

**Affiliations:** 1 Federal University of Pará (UFPA), Belém - PA, Brazil; 2 University of Münster, Münster, Germany; 3 University of Münster and University of Osnabrück, Osnabrück, Germany

**Keywords:** Socialization goals, Prosocial behavior, Spontaneous helping

## Abstract

The purpose of this descriptive-correlational study was to investigate possible associations between maternal socialization goals and prosocial behavior (spontaneous helping) among children living in a rural context. This study involved 39 dyads of mothers, aged 17 to 48 years (*M* = 24.28 years, *SD* = 5.97), and their children, with a mean age of 24 months. The data collection included a Sociocultural Sociodemographic Characterization Questionnaire (SSCQ), a Socialization Goals Questionnaire (SGQ), and a Spontaneous Prosocial Behavior Task (SPBT). Most importantly, we found that the maternal socialization goal to “Learning to support others” exhibited a significant correlation (*r =* 0.40, *p <* 0.05) with the helping task performed by the children. This finding sustains the hypothesis that maternal goals, which emphasize the importance of their children in learning to help others, are correlated with an increased frequency of prosocial behavior in young children, around their second birthday.

## Background

Parental socialization goals, regarding their children’s development, have gained a growing interest among researchers (Carra, Lavelli, Keller, & Kärtner, 2013; Kärtner, Keller, & Chaudhary, 2010; Keller & Kärtner, 2013; Ng, Tamis-LeMonda, Godfrey, Hunter, & Yoshikawa, 2012), who seek to comprehend the influence of socialization goals and parenting practices on a child’s developmental trajectory.

According to Keller, Lamm, Abels, Yovsi, Borke, & Jensen (2006) and Keller (2007), assumptions, socialization goals, and parenting experiences with primary caregivers are informed by features of the sociocultural context where they emerged and influence early child development. This study searches for differences between socialization goals and variables that describe the sociocultural context in which parents and their offspring live together. In other words and based on this basic premises (Keller, 2007; Keller et al., 2006), it is here assumed that socialization goals and parenting practices are closely tied to directions, child development might follow. Specifically, how far such goals and practices may affect patterns and frequency of child behaviors, is the main objective of this investigation.

Parental socialization has been researched systematically in many countries, with a literature replete of parenting beliefs and practices, as well as developmental paths (Jaramillo, 2012). Along these lines, Durgel, Leyendecker, Yagmurlu, and Harwood (2009) conducted their experiments, focusing on (a) exploring differences and similarities between the long-term socialization goals of German mothers and Turkish immigrant mothers living in Germany, and (b) analyzing the relationship between socialization goals of Turkish immigrant mothers’ and their cultural attitudes. All observed women, divided in two main groups, lived with their preschool kids in Germany. First group comprehended 79 mothers of Turkish ascendance, or that had migrated to Germany at a later age. The other group consisted of 91 German mothers. Turkish ascendance mothers exhibited more expectations from their children in maintaining close family ties and were less inclined to encourage autonomy than German mothers. Nevertheless, different situations were also verified. Turkish mothers who sympathized and were integrated into German culture emphasized individualistic ambitions (such as self-control), more often than a traditional/conventional Turkish woman.

This research relates to theoretical issues raised by Harkness and Super (1994) when they presented the Developmental Niche concept, whose preludes focus on a child’s relationship with the environment she/he is reared, educated, and protected, whether by the parents or other caregivers. According to Harkness and Super (1994), there is a complex structural model, with three subsystems in constant motion and interaction: the physical and social environment the child lives in (i.e., the family structure and form of social organization); parenting and schooling practices culturally imposed and regulated (intergenerational relations); and psychological traits of caregivers, comprising parental ethnotheories, which would include child-related beliefs and values, parenting goals, and strategies.

In this respect, analyzing parents and their beliefs can be viewed as an effective way of gaining access to various cultural models or their developmental implications. Thereby, it enriches our comprehension of mutual relationships between cultures and individuals (Macarini, Martins, Minetto, & Vieira, 2010). As conducted by Keller et al. (2006) and Keller (2007), researches concerned to mothers in various socioeconomic and cultural contexts (German, Euro-American, Greek, Indian, Chinese, Mexican, and Costa Rican) proposed three general models of parenting beliefs and values: autonomous, relational, and autonomous-relational. The first model relates to a unique and distinct self, prioritizing personal aspirations and autonomy, with beliefs developing for an autonomous self, whereby socialization goals are generally focused on self-realization and independence. The second model can be found in collectivist cultures, such as rural settings with a subsistence economy. It involves developing a self-linked to other individuals, viz., a self directly tied to the group he/she belongs. Here, socialization goals involve compliance with social norms, obedience, as well as acceptance of roles and hierarchies. In summary, the development of an interdependent self. The last model was described by Kağitçibaşi (1996), as a perspective that combines characteristics of first two models. This kind of cultural tendency involves both autonomy and interrelatedness, whereby the self is characterized as both autonomous, regarded to someone’s actions, and related, in terms of interpersonal distance (Keller, 2012; Keller et al., 2006; Lordelo, Roethle, & Mochizuki, 2012; Mendes & Cavalcante, 2014).

The difference between models that combine diverse tendencies has elicited the interest of many present-day researchers, along an empirical approach over importance of the context, comprehending one’s origins and their implications in child development. Research in this direction has been conducted on various cultures (Carra et al., 2013; Durgel et al., 2009; Harkness & Super, 1992; Ng et al., 2012) and socioeconomic contexts (Keller, 2007; Keller et al., 2006; Lordelo et al., 2012).

This paper attributes special attention to the second pattern of general trends, or interdependent model. Main features of this model are heteronomy and interrelationships, facets of a prototypical relational context characterized by subsistence agriculture, obedient behavior, and interpersonal compliance (Köster, Cavalcante, Carvalho, Resende, & Kärtner, 2016). Responsibilities are primary socialization goals (SGs), perceived as key indicators of social competence and optimal development. In these contexts, young children normally engage in daily tasks since an early age and, as they grow, more demanding tasks are assigned, such as taking care of younger siblings and housework. Many authors of behavior describe socialization via responsibility and involvement in household chores as a basis for prosocial conduct (Köster et al., 2016; Lancy, 2012; Ochs & Izquierdo, 2009).

Rural environments preserve traditional parenting practices, whereby mothers generally are the primary caregiver. Considering that, motherhood entails willingness to learn how to competently care of children, offer them love and affection, and to provide them with opportunities for their development. Regarding child socialization in relational contexts, mothers value mostly good manners and adaptation to social expectations, reaffirming respect and cooperation as important features for the social context and fulfillment of obligations, especially when these tasks are established within the family environment (Macarini, Martins, Sachetti, & Vieira, 2009).

Apart from children caring, mothers are also responsible for household chores, whereas men generally work outside home, caring for the family’s income (Ruela & Seidl-de-Moura, 2007). With so many responsibilities, mothers involve their children in household chores, encourage them, for example, to help with domestic issues, affording mothers a key role in developing prosocial motivation and conduct (Ochs & Izquierdo, 2009). This way, they reinforce involvement of their children in tasks, aimed the wellbeing of the other household members.

According to Keller (2012), from an early age (around 3–4 years), children that reside in a rural setting aid with household chores such as cleaning, fetching water and sticks, assistance in family business (for example, selling farm products or homemade foods), and babysitting younger siblings or other relatives. Following this theoretical assumption, a series of empirical studies have demonstrated that even very young kids exhibit enthusiasm to helping matters, providing/sharing available resources or information, even to strangers or individuals from whom these children receive no direct benefits (Costa & Cavalcante, 2012; Warneken, Hare, Melis, Hanus, & Tomasello, 2007; Warneken & Tomasello, 2006; Warneken & Tomasello, 2009).

Warneken and Tomasello (2009), regarding circumstances in which an adult inadvertently drops an object, e.g., a pen, reveal the same general tendency among children: they pick up the object after it falls on the floor. According to same authors (2006; 2007), children aged around 14 months start helping others in these circumstances, and such behaviors become increasingly frequent and sophisticated over time (i.e., they offer assistance in such situations and in other, more complex tasks).

Moreover, these researchers consider helping others as an extremely interesting phenomenon, in both cognitive and motivational sense. For cognitive terms, it is presumed that, to assist somebody, his/her intended objective and existing impediments to achieve it must be known by anyone who aims to help. Nonetheless, this can also be viewed in motivational terms, through the extent of efforts people apply to help others, even when there are no immediate gains for themselves. Therefore, it is inferred by the social interaction view (Dahl, 2015; Köster et al., 2016; Köster, Schuhmacher, & Kärtner, 2015) that natural proclivities to help are influenced by the social environment in which they occur.

There is now experimental evidence that infants understand individual needs, when they start to help others (Köster, Ohmer, Nguyen, & Kärtner, 2016) and also that their motivation to help is influenced by the way parents demonstrate helping behavior (Dahl et al., 2017), when others model helping behavior (Schuhmacher, Köster, & Kärtner, 2018), parenting practices of mothers (Torréns & Kärtner, 2017), and involvement of their children in daily shores (Köster et al., 2016). Parent and others behavior in such circumstances can thus effectively reinforce helpful behavior on children and encourage learning of prosocial norms.

Owing to interaction with others, children can prosocially evolve, being able to learn rules of sociability, develop open-mindedness, express their sentiments, and even control impulsive instincts. For Costa and Cavalcante (2012), as children employ their prosocial abilities, they tend to imitate normal activities observed in family members, allusive to different adapting manners, whose routines accord their particular environment. Considering Saud and Tonelotto (2005), prosocial conduct allows children not only to promote wellbeing, acceptance, and support for others but also to achieve, appreciate, and undergo socialization experiences, which develop their abilities and contribute to understand their attitudes and impacts on social interaction.

“Developmental Niche” considers that children develop through interaction of subsystems this theory comprises (Harkness & Super, 1992, 1994). Supported on such theoretical framework, this paper investigates maternal socialization goals and their role in child development, namely their influence on prosocial behavior in early childhood. Specifically, the present research examines potential correlations between maternal socialization goals and spontaneous prosocial behavior among children from a rural context in Brazil.

## Methods

### Participants

All data came from 39 mother-child dyads, aged 17 to 48 years (*M* = 24.28, *SD* = 5.97), with an average of 1.85 children (*SD* = 1) each; their children (53% male) had a mean age of 24 months (*SD* = 3.7).

With respect to school level, 66.7% of the mothers had a high school education. About 53.8% reported not working outside home, dedicating exclusively to domestic activities. In such cases, it would be expected that mothers encourage their children to take part of family activities from an early age on. Somehow, children have to partake of daily chores, such as fetching or carrying things. The 66.7% mentioned mothers stated that they dedicate at least 12 h a day to their children, and 84.6% reported dedicating even more time. Regarding birthplace, 89.7% of analyzed children reside at their mother birthplace in Castanhal municipality.

### Context of the study

Agricultural communities compose the rural context analyzed in this research. They were agricultural villages of Amazon region, near Belém city (more precisely, villages of Boa Vista, Pacuquara, Itaqui, and Santa Teresinha). There, settlements with 50 to 300 families were identified, living in simple houses made of wood, or brick stones. Mothers were mostly housewives, while fathers were mainly engaged in the agricultural sector, like cultivation and processing of local plants. Material possessions were few. However, the villages were connected to the power system. Many houses had television. Some daily bus services connected the villages to urban areas of Castanhal (approximately 30–60 away). Most children received basic education at local schools (Köster et al., 2016).

### Environment

Preserving daily surroundings of dyades, data samples of this research considered settings where mothers and children lived together, i.e., both resided and manifested presence at the same home. Before answering questionnaires, each mother was requested to select one room of their homes, in which a table and a chair were available, for conducting the survey.

### Instruments and materials

Data was collected via three instruments.

#### Sociocultural-sociodemographic characterization questionnaire

Questionnaire employed for sociodemographic variables (family structures and schooling levels of caregivers) is the same of earlier studies, conducted by Vieira et al. (2010). It comprises 25 questions about sociodemographic themes, concerning parents/caregivers (age, schooling, occupation, birthplace, raising locations) and children (age, sex, and birthplace). The following questions were included: How often the child interacts with their father per day (an average number)? How often the child interacts with their mother per day (an average number)? How many people live into the house? How many siblings does the child have? (Answers must provide details of child’s birth, current age, and sex).

#### Socialization goals questionnaire

Maternal socialization goals were assessed through structured interview, with questions adapted from a questionnaire elaborated by Kärtner et al. (2010). Its approaches (a) an autonomous socialization goals scale, related to child’s self-confidence and assertiveness (11 items); and (b) relational socialization goals scale. This one comprehends two subscales, namely prosocial behavior (11 items) and obedience (7 items). Mothers were requested to indicate, through a four-point Likert scale (strongly disagree, slightly disagree, slightly agree, and completely agree), a degree of agreement with socialization goals surveyed in the questionnaire (Cronbach’s *a* > .76).

#### Spontaneous prosocial behavior task

Spontaneous instrumental helping behavior in children was based on an adapted version of an out-of-reach task, developed by Warneken and Tomasello (2006): using clothespins, the experimenter (E1) begins hanging three towels. For each towel, E1 drops a clothespin, reaches for it, and tries to grasp it, without success. While trying to retrieve the object, E1 keeps his/her eyes on the clothespin (for 30 s), then alternates his/her glance between the towel and the child (for 30 more seconds), and finally addresses the child, calling out the child’s name twice (final 30 s). If the child offers no help, E1 picks up the clothespin and continues with the next towel.

#### Procedures and ethical considerations

Study protocol and data collection was approved by Research Ethics Committee of Tropical Medicine Nucleous from Federal University of Pará (number 059/2011 CEP/NMT). Throughout data gathering, two experimenters conducted the task sessions at each family’s home. The participating mothers signed consent forms, agreeing with the aims of the experiment and essential procedures. Furthermore, the selected observation method involved minimal interference in the environment, reducing risks to physical and emotional integrity, whether for mothers or children. Moreover, a familiarization period among researchers and participants was established, in order to avoid potential threats against family members.

Spontaneous interaction between mothers and their children inside the household environment (implementation method, storage, and purpose) was severely considered. First experimenter (E1), research assistant, and child played with an ordinary toy for 10–15 min. Next, E1 administered the sociodemographic and socialization goals questionnaires, as conducted the spontaneous prosocial behavior tasks. Second experimenter (E2) prepared behavioral assessments, filming actions performed both by mother and child during the tasks.

### Data analysis

The statistical analyses were conducted using the PASW Statistics for Windows version 20.0 software (SPSS, Inc. Chicago, IL, USA). To describe sociodemographic variables and socialization goals, standard deviations with percentages and means were calculated. Maternal socialization goals were grouped accord Socialization Goals Questionnaire, and self-models discriminated by Keller et al. (2006) and Keller (2007). Next procedures considered inclination of mothers for autonomy and interdependence. Yet, spontaneous prosocial behavior was also described. Frequencies and percentages calculations indicated degree of child’s hesitancy in performing a proposed task. Finally, acquired data were crossed, so maternal socialization goals and children’s prosocial behavior could be statically correlated (Pearson correlation).

## Results

The present section is organized in three parts. Part I: the results for maternal socialization goals. Part II: describes children’s behavior inside research environment, i.e., their completion of (or failure to) complete tasks. Part III: reveals how previous parts, as their features, are correlated.

### Maternal socialization goals

There is prevalence of relational socialization goals (*M* = 3.34, *SD* = 0.46) over autonomous socialization goals (*M* = 2.53, *SD* = 0.58). Over 50% of mothers agreed with socialization goals associated to relational self-model, such as *learning to respect seniors* (74.4%), *learning to obey seniors* (69.2%), *learning to obey parents* (69.2%), *learning not to contradict parents* (56.4%), *learning to do what parents say* (56.4%), *learning to give things to others* (53.8%), and *learning to help others* (51.3%). Furthermore, a large number of mothers completely disagree with the following goals: *learning to occupy oneself on oneself* (30.8%), *developing independence* (28.2%), *learning to be independent* (28.2%), *learning to make decisions* (25.6%), and *learning to distinguish oneself in one*’*s group* (20.5%).

### Spontaneous prosocial behavior task

About 51.27% of observed children immediately performed the proposed tasks. Against 23% who did not complete the task (see Table 1). By comparing these results with children’s ages, it was noticed that the youngest children (18–21 months) were less propitious to perform the task immediately (7.69%); oldest children (26–30 months) offered spontaneous help; the median age group children (22–25 months) were more persistent, with low percentage in quitting task, despite their incomplete results (see Table 1).

**Table 1:** Table of the children’s performance, in spontaneous prosocial task

Age (months)	*n*	Immediately		Slightly hesitant		Very hesitant		Task not completed	
		*N*	%	*n*	%	*n*	%	*n*	%
18–21	14	3	7.69%	3	7.69%	2	5.12%	6	15.38%
22–25	11	7	17.94%	1	2.56%	2	5.12%	1	2.56%
26–30	14	10	25.64%	2	5.12%	0	–	2	5.12%

#### Maternal socialization goals and children’s performance in executing the spontaneous prosocial behavior task

Data support a significant correlation between the socialization goals learning to help others (mother, siblings) and learning to respect and obey seniors (*r* = .74, *p* < 0.01)—see Table 2.

**Table 2:** Table of correlations between maternal socialization goals and the children’s performance of the spontaneous prosocial behavior task

Variables	1	2	3	4	5	6	7	8	9	10	11	12	13	14	15	16	17	18	19	20
1. Result of the task performed by the child	–																			
2. Learning to share with others	.22	–																		
3. Learning to support others	.40*	.42**	–																	
4. Learning to understand others’ feelings	.18	.27	.54**	–																
5. Learning not to manifest bad behavior	.21	.38*	.50**	.38*	–															
6. Learning to give some things to others	− .12	.28	.25	.41**	.69**	–														
7. Learning to obey parents	−. 16	.27	.37	.38*	.56**	.68	–													
8. Learning to take care of others	.11	.38*	.67**	.53**	.23	.20	.19	–												
9. Learning to obey seniors	− .24	.38*	.19	.20	.34*	.46**	.65**	.19	–											
10. Learning to care for others’ wellbeing	.05	.17	.59**	.56**	.34*	.35*	.48**	.62**	.38*	–										
11. Learning to comfort others	.13	.09	.49**	.54**	.35*	.40*	.35*	.63**	.13	.54**	–									
12. Learning to help others (mother, siblings)	− .01	.17	.42**	.37*	.31	.38*	.64**	.27	.74**	.46**	.34*	–								
13. Learning to cheer up others	− .18	− .12	.25	.19	.27	.37*	.44**	.05	.39*	.37*	.21	.55**	–							
14. Learning to participate when something needs to be done	.22	.04	.48**	.56**	.45**	.43**	.41**	.41**	.30	.37*	.61**	.65**	.40*	–						
15. Learning not to contradict one’s parents	− .00	.12	.13	.13	.31	.33	.30	− .04	.22	.04	.13	.11	.24	.31	–					
16. Learning to help others when they are sad or distressed	.21	.23	.55**	.31	.65**	.42**	.53**	.38*	.27	.49**	.57**	.45**	.36*	.62**		–				
17. Learning to behave according to social norms	.06	− .06	.32	.36*	.24	.20	.37*	.24	.16	.31	.12	.26	.24	.39*	.27	.17	–			
18. Learning to respect seniors	− .09	.29	.41**	.31	.41**	.51	.69**	.11	.71	.40*	.07	.64**	.45**	.32	.22	.19	.35*	–		
19. Learning to do things with others	.15	.12	.31	.38*	.37*	.37*	.13	.41**	.07	.44**	.56**	− .04	− .01	.32	.26	.34	.28	.04	–	
20. Learning to do what parents say	.10	.25	.35*	.16	.28	.23	.33*	.36*	.11	.07	.24	.18	.06	.32	.38*	.38*	.36*	.06	.28	–
M	–	3.19	2.86	2.84	3.11	3.49	3.65	2.51	3.62	2.70	3.16	3.38	3.35	3	3.27	3.27	2.81	3.68	3.27	3.46
SD	–	1	0.77	1	0.87	0.55	0.53	0.98	0.63	0.74	0.76	0.72	0.75	0.74	1	0.65	0.81	0.58	0.73	0.65

All coefficients are significant at *p* < .05

Among all the correlations tested, it was found that the goal of maternal socialization “Learning to support others” has a significant correlation with the child’s performance (*r =* .40, *p* < 0.05)—see Table 2.

## Discussion

A high percentage of analyzed children, around 89.7%, live at their mother’s birthplaces, Castanhal municipality. Most mothers were born and live in rural settings, according to Ruela and Seidl-de-Moura (2007). Namely, they tend to be connected to their family of origin. In this sense, family culture, as well as their beliefs, values, or traditions, are deep-rooted and handed down along different generations. Hence, for mothers in the present study, it may be presumed that the characteristics of their environment (e.g., birthplace and current residence location) were transmitted from generation to generation in a more continuous manner, with less interference from other cultures.

Regarding maternal age range (17–48 years), educational level and number of children reported by mothers, expose a context which, though typically rural, is passing through a transitional phase with different models of cultural tendencies (from relational to autonomous-relational), as previously described by Keller et al. (2006) and Keller (2007). In relational contexts, mothers tend to have lower schooling levels than those reported in the present study, given 38.5% of them stated to pursue an incomplete high school education. Similarly, rural women generally have many children. However, almost half of the mothers in our sample (46.2%) reported having only one child. This is extremely significant because some of current researchers believe that a mother’s educational level and occupation is directly related to her notions concerning child development. Lima, do Nascimento, and da Silva Farias (2016) associate transition to globalization and modernization, new consumer trends, and flexibility of job market (commerce, farming). From this standpoint, it is possible to suppose that, at macro-socio-historical level, such factors are progressively transforming Brazilian family attitudes and, consequently, parent/caregiver notions of both child development and rearing.

Another noteworthy point is that around 54% of mothers stated their activities center on their homes, typical occupation of women in traditional rural communities. In such circumstances, women basically occupy themselves with household chores and childcare, whereas men work outside their homes (in farming and commerce). This family arrangement can partly explain why mothers here accounted spend most of their time rearing their children, given 66.7% reported 12 h of dedications a day on weekdays, and 84.6% on weekends. This variable is significant, in view of the fact that it is generally acknowledged the longer a mother interacts with her child, the greater the possibility of influencing child with her beliefs, values, and childcare practices.

Based on principal assumptions of Keller (2007), once mothers establish different socialization goals for their children, it might explain why some mothers are inclined to prefer certain goals compared to other, based on sociocultural characteristics of their environments. Accordingly, and considering that this investigation was conducted in rural surroundings, the hypothesis here defended is that participating mothers would prioritize goals focused on importance of social roles, duties, and obligations favoring their groups. Moreover, socialization goals and proceeded correlations point to certain direction (Keller, 2007; Keller et al., 2006; Lordelo et al., 2012), namely obedience to older people—and thus to individuals who typically enjoy superior hierarchical status—which is of great value in rural communities and environments.

Another point that deserves emphasis regards to observations of the spontaneous prosocial behavior task performed by children. Results revealed that older children achieved higher scores than younger ones. Along these lines, and supplementing recent studies (Kärtner et al., 2010; Keller, 2012; Warneken & Tomasello, 2006, 2007) which assert children begin helping with household chores since a very young age, the present research provides evidence that a child’s spontaneous prosocial behavior becomes increasingly sophisticated and frequent over time, which may be attributable not only to biological traces but also to parental encouragement in situations of helping behavior, where parents praise attitudes of mutual benefits (Dahl et al., 2017; Köster et al., 2016).

In addition to the results of significant correlation between spontaneous prosocial behavior and a child’s age, another accentuated correlation combining typically rural maternal socialization goals and prosocial behavior manifested by children inside their context was confirmed. This happens because rural mothers maintain loaded household chores. Thereby, children must socialize and partake in different tasks in order to relieve such load (cf. Köster et al., 2016).

## Conclusions

Our findings sustain the hypothesis that mother goals and day-to-day practices—that enhance importance of teaching kids in helping others—are associated to the frequency of helping behavior on children. This fact supports that both child’s involvement and mother’s encouragement play an important role on child’s prosocial development.

According to current literature, we expected mothers living in rural environments to exhibit typical relational model traces, involving heteronomy, relationships, and cooperation among family members, as well as other contextual characteristics such as minimal formal education and early reproduction. In contrast, most of the surveyed mothers had a moderate education and small families, with only one child each. Thus, we conclude the agricultural villages in the Apeú-Castanhal district are characterized by prevalence of a relational cultural model, despite an observed transition to an autonomous-relational model.

We further conclude that the present study would be especially valuable for academic counseling activities, both theoretical and practical. In a theoretical scope, it would provide material for research, pondering mother’s cogitation for children’s future, traditional community values, sociocultural features, and prototypical contexts.

Such shortcomings reflected the limited number of dyads we surveyed, as well as a transitional context, with models of cultural tendencies in constant changes. At present, we perceive the need to conduct further similar studies at larger scales, and to examine environments of even more traditional populations, such as *quilombola* communities and riparian villages.

## Abbreviations

SGQ: Socialization Goals Questionnaire
SGs: Socialization Goals
SPBT: Spontaneous Prosocial Behavior Task
SSCQ: Sociocultural Sociodemographic Characterization Questionnaire
